# Morphological Characteristics and Habitats of Red Seaweed *Gracilaria* spp. (Gracilariaceae, Rhodophyta) in Santubong and Asajaya, Sarawak, Malaysia

**DOI:** 10.21315/tlsr2018.29.1.6

**Published:** 2018-03-02

**Authors:** Muhammad Nur Arif Othman, Ruhana Hassan, Mohd Nasarudin Harith, Amir Shah Ruddin Md Sah

**Affiliations:** 1 Department of Aquatic Science, Faculty of Resource Science and Technology, Universiti Malaysia Sarawak, 94300 Kota Samarahan, Sarawak, Malaysia; 2 School of Biological Sciences, Universiti Sains Malaysia, 11800 USM Pulau Pinang, Malaysia

**Keywords:** Sarawak, *Gracilaria*, Morphological Approach, Water Quality, Habitat

## Abstract

Red seaweed *Gracilaria*, one of the largest genus in Division Rhodophyta inhabits Sarawak coastal water. This study was designed to identify the species of *Gracilaria* using morphological approach and to assess selected water quality parameters in *Gracilaria* habitats. Three field samplings were carried out in Santubong and Asajaya, Sarawak from November 2013 to December 2014. Overall, three species were identified namely *Gracilaria changii*, *G. blodgettii* and *G. coronopifolia*, attached to net of cage culture in Santubong and root of mangrove trees in Asajaya. In addition, three different taxa of aquatic macroinvertebrates (polychaete, small crab, bivalve) and single species of red seaweed (*Acanthophora* sp.) were observed in *Gracilaria* assemblages. An estimate of 37% to 40% of the upper part of the cage net in Santubong was covered by seaweeds and only 16% to 20% in Asajaya’s mangrove. The study had provided better information on identification of *Gracilaria* and their habitat in Sarawak. Future work involving DNA barcoding of each species is in progress.

## Abstract (ru)

Rumput laut merah *Gracilaria*, salah satu genus terbesar bagi spesies Rhodophyta yang mendiami pesisir pantai Sarawak. Kajian ini dijalankan untuk mengenal pasti spesies *Gracilaria* menggunakan pendekatan morfologi dan untuk menilai parameter kualiti air terpilih dalam habitat *Gracilaria*. Tiga pensampelan lapangan dijalankan di Santubong dan Asajaya, Sarawak dari November 2013 hingga Disember 2014. Secara keseluruhannya, tiga spesies telah dikenal pasti iaitu *Gracilaria changii*, *G. blodgettii* dan *G. coronopifolia*, yang tersangkut pada jaringan sangkar di Santubong dan akar pokok bakau di Asajaya. Di samping itu, tiga jenis makroinvertebrata akuatik (*polychaete*, ketam kecil, dwicangkerang) dan spesies tunggal rumput laut merah (*Acanthophora* sp.) diperhatikan dalam himpunan *Gracilaria*. Anggaran 37% hingga 40% daripada bahagian atas sangkar di Santubong dilindungi oleh rumpai laut dan hanya 16% hingga 20% di bakau Asajaya. Kajian ini telah memberikan maklumat yang lebih baik mengenai pengenalan *Gracilaria* dan habitat mereka di Sarawak. Kajian masa depan yang membabitkan pengkodan DNA bagi setiap spesies juga sedang dijalankan.

## INTRODUCTION

*Gracilaria* is one of the Genus in Family Gracilariaceae with more than 100 species worldwide, inhabiting temperate and tropical seawaters, covering from intertidal to subtidal areas (Food and Agriculture Organisation (FAO) (1990); Gulbransen *et al.* (2012)). *Gracilaria* is important as source of income in country such as Chile, where they have been cultured commercially with total landings of 120,000 wet metric tons (Buschmann *et al.* 2001). In Sarawak, Malaysia, wild *Gracilaria* are collected by the local people as source of food and generate income by selling them in market (Othman *et al.* 2015). From ecological aspect, *Gracilaria* act as natural habitat for aquatic organisms and protect them from predators, waves and tides (Nyberg *et al.* 2009) and certain fishes, crabs and isopods prefer *Gracilaria* as food (Veeragurunathan *et al.* 2014).

In Sarawak, effort has been done to document the seaweed resources from coastal waters (Ariffin 2006; Esa 2014; Jusoh 2012; Nurridan 2007; 2013; Wong *et al.* 2012; Zakaria *et al.* 2006; Zawawi *et al.* 2015). According to Nurridan (2007), there are about 87 species of marine seaweeds found along the coastal water of Sarawak, belongs to 27 species of Division Chlorophyta, 21 species of Division Phaeophyta and 39 species of Division Rhodophyta. Up to now, there are 10 species of *Gracilaria* recorded in Sarawak namely *Gracilaria arcuata, G. articulata, G. changii, G. coronopifolia*, *G. blodgetti, G. salicornia, G. edulis, G. textorii* and two unidentified species (Nurridan 2013).

*Gracilaria* is an ambiguous species where misidentification often occur due simple morphology and high plasticity problems (Phang *et al.* 2001; Saunders 2009). Besides, the different features of male and female in same species as well as variation in morphologies of their life cycle also could cause difficulty during identification (Baghel *et al.* 2011; Robba *et al.* 2006). In Sarawak, the identification of *Gracilaria* was done by Nurridan (2007; 2013) and the study should be continued to resolve the taxonomy and create clear understanding on their morphology. Besides, the available studies of seaweed in Sarawak focusing on the species checklist and the habitat information especially *Gracilaria* is limited. Therefore, the objectives of this study were to: (i) describe the morphological characteristics of *Gracilaria* species found in Santubong and Asajaya, Sarawak and (ii) assess selected water quality parameters in *Gracilaria* habitats as well as the aquatic organisms associated with it.

## MATERIALS AND METHODS

**Figure 1:**
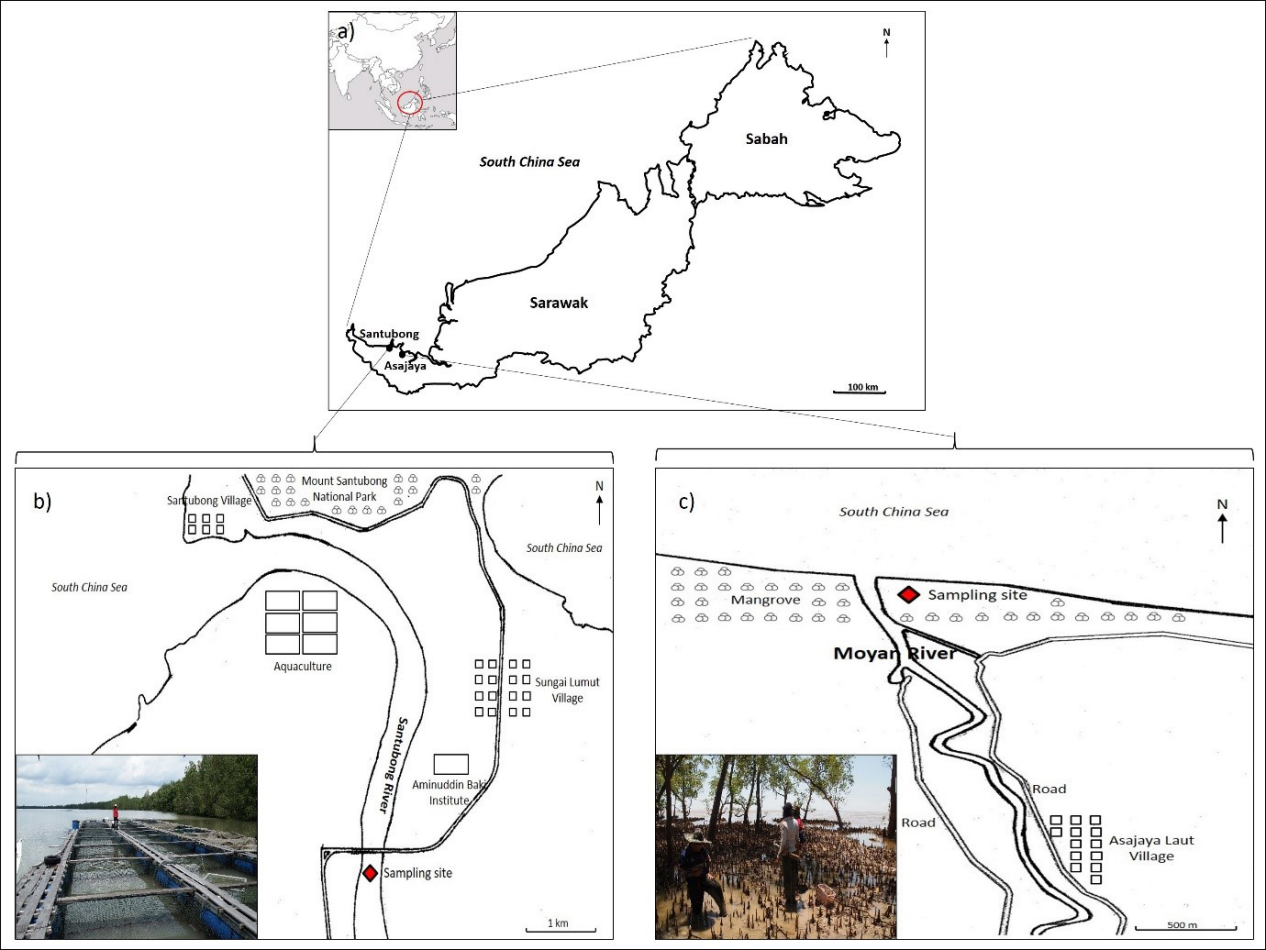
Field samplings area in Santubong and Asajaya, Sarawak.

Three field samplings were carried out in cage culture area, Santubong, Sarawak (N 01° 40′42.1″ E 110° 20′2.4″) and mangrove area, Asajaya, Sarawak (N 01° 35′57.8″ E 110° 36′15.9″) (Fig. 1). For Santubong, the samplings were done during flooding tide while for Asajaya, the samplings were done during ebbing tide. All the samplings were done at daylight. The descriptions of the sampling sites are shown in Table 1.

Selected water quality parameters namely dissolved oxygen (DO), temperature, turbidity, pH, conductivity and salinity were measured *in-situ*. DO and temperature were measured using Hanna instrument (model 9142). Turbidity was taken using Eutech instrument (model TN-100). Milwaukee instrument was used to record salinity while Hanna instrument (model HI 8424) was used to take pH reading. Other parameters such as water transparency was recorded using secchi disk while the depth of water was measured using depth finder (Speedtech instrument, model 65054). Triplicate readings of all the water quality parameters were recorded. Four litres of water samples were collected using acid wash polyethylene bottles for nutrient (nitrite, orthophosphate, silicate), chlorophyll *a* (chl *a*) and total suspended solid (TSS) analyses. In Santubong, the water samples were collected at the water surface during flooding tide while in Asajaya, the water samples were collected at water puddle during ebbing tide. The samples were kept in cooler box with ice and brought back to laboratory in Universiti Malaysia Sarawak (UNIMAS) for further analysis. Chl *a* analysis followed the method proposed by Aminot and Rey (2000), TSS analysis followed the method proposed by Jacobs Engineering Group (2010) and nutrients analysis followed the standard protocol Hach (2007) using spectrophotometer (Hach, DR 2010). The values obtained were compared with Malaysian Marine Water Quality Criteria and Standard (MMWQCS) (Department of Environment (DOE) 2010). In Asajaya, 30 g of subsurface sediment was collected using scope for particle size analysis. The samples were collected in triplicates, stored in plastic bag with appropriate labels and brought back to laboratory for further analysis. Sediment particle size was analysed using method proposed by Buchanan (1984). One-way analysis of variance (ANOVA) was performed to test the significant difference of water parameters among field samplings in each location. The test was significant at *p* < 0.05.

The whole thallus of 15 *Gracilaria* individuals were collected at cage culture in Santubong and mangrove area in Asajaya consists of holdfast, blade and stipe. The samples were washed, stored in plastic bag with zipper and brought back to laboratory for further analysis. Other organisms associated with *Gracilaria* were also observed, collected, stored in plastic bag and brought back to laboratory for identification. At the lab, the *Gracilaria* samples were preserved using wet preservation and dry preservation, methods suggested by Dhargalkar and Kavlekar (2004). The other organisms were preserved in 10% formalin. Identification of *Gracilaria* species and other seaweed were based on the identification keys from Nurridan (2007; 2013), Dhargalkar and Kavlekar (2004), Ismail (1995) and Lin (2009).

Percentage coverage of seaweeds were observed in four cages of Santubong cage culture using modified grid transparency quadrate (0.058 m^2^). In December 2014, a 100 m transect line was set up parallel to the seashore in Asajaya and a quadrate (0.25 m^2^) was randomly throw for every 25 m to determine the percentage coverage of seaweeds.

## RESULTS AND DISCUSSION

A total of three *Gracilaria* species were found in Santubong and Asajaya, Sarawak namely *G. changii*, *G. blodgettii* and *G. coronopifolia*. Table 2 showed the voucher list of the seaweed specimens that were deposited in the Department of Aquatic Science Museum (Botanical Section), UNIMAS. Summary of their morphological characteristics shown in Table 3.

### *G. blodgettii* Harvey, 1853

#### Synonyms

None

#### Taxonomy Remarks

**Figure 2:**
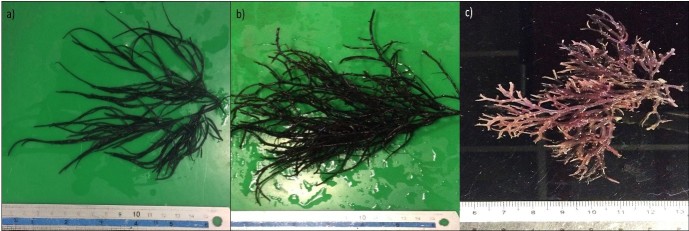
(a) *G. blodgettii,* (b) *G. changii,* (c) *G. coronopifolia.*

*G. blodgettii* had a dark red colour and the thallus could grow up to 200 mm tall (Fig. 2a)*.* The holdfast shape was discoid while the branch shape was cylindrical with diameter between 1 to 2 mm. Primary branches were longer compare to secondary branches and could reach up between 10 mm to 90 mm long while secondary branches could reach up to 5 mm to 85 mm. The branches either secund or irregular, slightly constrict at the base, enlarged at the middle and become attenuate at the tip. Frequent formation of branches was observed at secondary branches which make this species look compact. New formation of short branches with pointed tip were observed at tertiary branches. The cross section of stipe show that the medulla was composed of 3–4 layers of parenchymatous cells and surround by 2–3 layer of small rounded cortical cells at the cortex.

#### Location

Asajaya, Sarawak

#### Ecology and Distribution

The specimen was found attached to root of mangrove trees in Asajaya, Sarawak. This finding was similar to report by Nurridan (2007). Other than Asajaya, *G. blodgettii* was also found in Muara Mengkuang, Miang Kecil, Sungai Sibu, Salak, Kuching Division.

### *G. changii* (Xia & Abbott) Zhang & Xia, 1991

#### Synonyms

Polycavernosa changii (Xia & Abbott), Hydropuntia changii (Xia & Abbott) (Wynne, 1989)

#### Taxonomy Remarks

the colour of *G. changii* was dark red while the thallus could grow between 180 mm to 220 mm tall (Fig. 2b). Primary branches were shorter compare to secondary branches and could reach up between 25 mm to 40 mm long while secondary branches could reach up to 40 mm to 170 mm. The species had discoidal holdfast and the branches were irregular with diameter between 1 to 2 mm. Constriction occur at base of branches, swelling at middle and tapering toward the end. The formation of branches occurs occasionally. The tip of secondary branches either pointed or divide into two short branchlets. Formation of new branches with pointed tip were observed along tertiary branches. The cross section of stipe show that the medulla was composed of 3–4 layers of parenchymatous cells and surround by 2–3 layer of small rounded cortical cells at the cortex.

#### Location

Santubong, Sarawak

#### Ecology and Distribution

The specimen was found attach to net of cage culture in Santubong, Sarawak. Nurridan (2007) reported that *G. changii* attach to net, buoys and floating net cages. *G. changii* could be found in other areas of Sarawak namely Pulau Salak, Kuching Division. In Peninsular Malaysia, *G. changii* was found in Morib, Selangor (Chan *et al*. 2002). *G. changii* can also be found in Malacca, Penang, Selangor, Negeri Sembilan, Johor, Kedah, and Sabah.

### *G. coronopifolia* J. Agardh, 1852

#### Synonyms

G. lichenoides f. coronopifolia (J. Agardh) (May, 1948)

#### Taxonomy Remarks

*G. coronopifolia* had a purplish-red colour (Fig. 2c). The species had discoid holdfast, cylindrical and irregular branches with diameter between 1 to 2 mm. shorted pointed tip, frequent formation of branches were observed at the end of thallus where each subsequent branching was shorter than previous branches. Upper part of thallus more densely branches and form small bush. The branches had no constrict base and tapering toward the end. The last branches were bifurcate. The cross section of stipe show that the medulla was composed of 3–4 layers of parenchymatous cells and surround by 1–2 layer of small rounded cortical cells at the cortex.

#### Location

Santubong, Sarawak

#### Ecology and Distribution

The specimen was found attach to net of cage culture in Santubong, Sarawak. Nurridan (2007) reported that *G. coronopifolia* attach to net and buoy at floating net cage system. *G. coronopifolia* could be found in other areas of Sarawak namely Pulau Salak, Kuching Division.

Based on Table 4, the surface water temperature in Santubong had range of 28.80°C–29.90°C [F (2,6) = 273.00; *p* = 0.000] while Asajaya had range of 29.30°C–31.37°C [F (2,6) = 70.26; *p* = 0.000]. Santubong had recorded pH with range of 6.60–7.82 [F (2,6) = 16.12; *p* = 0.004] while Asajaya had recorded pH with range 7.41–7.80 [F (2,6) = 83.27; *p* = 0.000]. In Santubong, the pH probably affected by the decomposition of foods and waste products that come from culturing activities (Suratman *et al.* 2014). For Asajaya, the water pH possibly affected by photosynthesis rate where the presence of *Gracilaria* used the CO_2_ for photosynthesis process thus increasing the pH (Tucker & D’Abramo 2008).

Santubong had recorded DO with range of 5.29 mg/L – 6.69 mg/L [F (2,6) = 19.00; *p* = 0.003] while Asajaya had recorded DO with range of 4.05 mg/L – 5.68 mg/L [F (2,6) = 6.36; *p* = 0.033]. Noraini *et al*. (2010). reported that abundance of aquatic plants could increase the water DO where the presence of *Gracilaria* and other type of seaweeds could be observed in Santubong and Asajaya. Santubong had recorded salinity with range of 20.33 PSU – 28.33 PSU) [F (2,6) = 228.50; *p* = 0.000] while Asajaya had recorded salinity with range of 18.33 PSU – 23.44 PSU [F (2,6) = 9.93; *p* = 0.013]. Water salinity in Asajaya and Santubong could be affected by tidal change, evaporation and mixing of freshwater and seawater (Wong *et al.* 2012; Coelho *et al.* 2007).

The turbidity in Santubong had range of 10.17 NTU – 35.10 NTU [F (2,6) = 259.74; *p* = 0.000] while Asajaya had range of 36.63 NTU – 253.33 NTU [F (2,6) = 24.73; *p* = 0.001]. In Santubong, the water turbidity could be affected by water discharge from human settlement areas, aquaculture activities along the Santubong river and soil erosion due to construction at the upper part of the river. The turbidity in Asajaya was high with range of 37.63 NTU – 253.33 NTU because the readings were taken during low tide where the suspended solid had been brought by the turbid river and deposited at the mangrove area thus increasing the turbidity reading. Due to technical problems, the depth and transparency readings in Santubong were only taken during the third sampling (October 2014). The depth and transparency readings for Santubong were 7.80 m and 0.73 m respectively.

Based on Table 5, Santubong had recorded TSS with range of 27.33 mg/L – 63.33 mg/L [F (2,6) = 2.04; *p* = 0.211] while Asajaya had recorded TSS with range of 161.10 mg/L – 655.57 mg/L [F (2,6) = 9.24; *p* = 0.015]. The TSS of Santubong river is possibly influenced by water discharge from residential areas, aquaculture and industrial activities. TSS in Asajaya was high with range of 161.10 mg/L – 655.57 mg/L because the readings were taken during low tide where the turbid river brings the particles such as silt, clay, sand, organic and inorganic matter from the upstream to the mangrove areas, trapped by the mangrove roots and deposited in the mangrove areas.

Santubong had recorded nitrite (NO_2_) with range of 0.012 mg/L – 0.046 mg/L C [F (2,6) = 27.00; *p* = 0.001], orthophosphate (PO_4_^3−^) with range of 0.050 mg/L – 0.063 mg/L [F (2,6) = 0.12; *p* = 0.888] and silicate (SiO_2_) with range of 0.579 mg/L – 3.509 mg/L [F (2,6) = 78.36; *p* = 0.000]. Asajaya had recorded NO_2_ with range of 0.005 mg/L – 0.015 mg/L [F (2,6) = 78.00; *p* = 0.000], PO_4_^3−^ with range of 0.057 mg/L – 0.173 mg/L [F (2,6) = 20.77; *p* = 0.002] and SiO_2_ with range of 1.560 mg/L – 2.740 mg/L [F (2,6) = 214.06; *p* = 0.000]. The runoff of sewage from residential areas, waste products from industrial activities with addition of aquaculture activities could influenced the nitrite and orthophosphate concentration in Santubong river. Leach of fertilizers and pesticides from the farms own by Asajaya’s local people may indirectly affected the nutrients. Santubong had recorded mean chl *a* reading with range of 2.556 mg/m^3^ – 7.663 mg/m^3^ [F (2,6) = 21.44; *p* = 0.002] while Asajaya had recorded chl *a* with range of 3.236 mg/m^3^ – 5.937 mg/m^3^[F (2,6) = 2.72; *p* = 0.145].

Table 6 showed the comparison of water quality results in Santubong, Sarawak with Malaysian Marine Water Quality Criteria and Standard (MMWQCS) values. The range of DO for all field samplings in Santubong was higher compared to the standard value set for Marine life, fisheries, coral reefs, recreational and mariculture (Class 2). The mean TSS in October 2014 was lower than Class 2 of MWQCS whereas the mean TSS in November 2013 and March 2014 were higher than standard value. the range of NO_2_ and PO_4_^3^ for all field samplings were lower compared to the standard value of Class 2.

Table 7 showed the comparison of water quality results in Asajaya with Malaysian Marine Water Quality Criteria and Standard (MMWQCS) values. The range of DO and TSS for all field samplings were higher compared to the standard value set for mangrove estuarine and river mouth water (Class E). In Comparison, the range of NO_2_ for all field samplings were lower compared to the standard value set for mangrove estuarine and river mouth water (Class E). the mean PO_4_^3^ in March 2014 was lower than Class E of MWQCS whereas the mean PO_4_^3^ in May 2014 and December 2014 were higher than standard value.

Based on the observation at the field, *Gracilaria* in Asajaya prefer root of mangrove trees compare to muddy sediment as their substrate. In contrast, *Gracilaria* found in Santubong river grow well by attaching themselves to the cage culture net which is a man-made structure. In Asajaya, the sediment comprised of high silt and clay contents (58.5%–65.8%) compare to sand (34.2%–41.5%) which make the substrate loosely arranged (Table 8). This probably the reason for *Gracilaria* to grow on more stable and strong mangrove roots that can protect them from incoming waves and prevent from swept away by the water current during flooding and ebbing tides. Based on studies done by Molloy and Bolton (1995), the type of sediment did influence the distribution of *Gracilaria*.

In Santubong, the estimated percentage cover of seaweeds on the cage net in October 2014 is 37% to 40% meanwhile the estimated percentage cover of seaweeds in Asajaya mangrove (December 2014) is 16% to 20%. No information on seaweeds percentage cover collected on first and second samplings due to technical problem. *Gracilaria* in Santubong cage culture were found living in patchy throughout the three samplings and similar case also was observed in Asajaya mangrove area. The actual percentage cover of *Gracilaria* in both sampling sites could not be obtained because the *Gracilaria* existed there probably had been collected regularly by the local people for personal consumption and selling for extra income.

**Figure 3:**
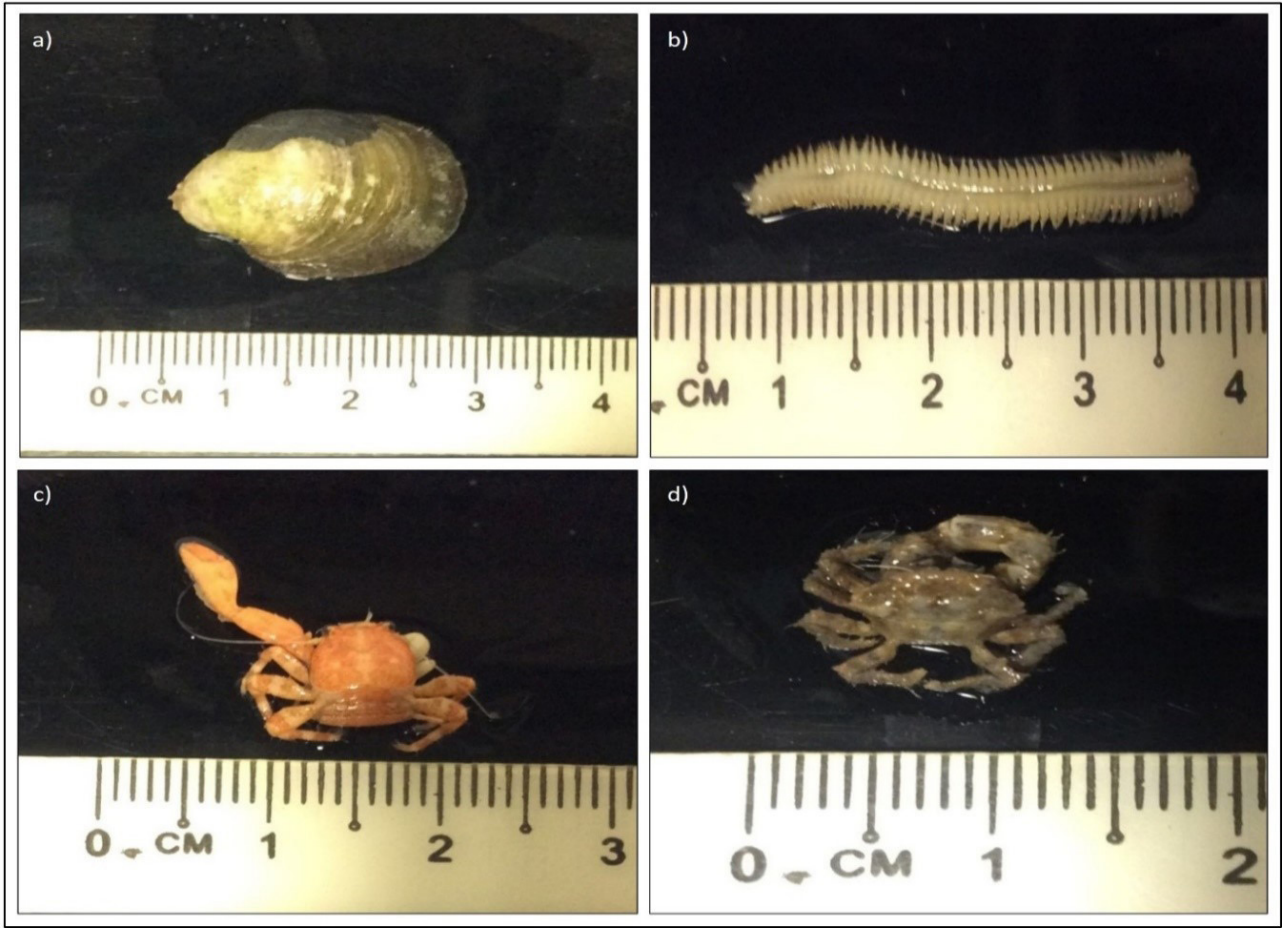
Aquatic macroinvertebrates (polychaete, small crab, bivalve) found living together with *Gracilaria* assemblages in Santubong.

Based on Fig. 3, three different taxa of aquatic macroinvertebrates (polychaete, small crab, bivalve) and single species of red seaweed (*Acanthophora* sp.) found living together with *Gracilaria* assemblages in Santubong (Fig. 3). According to Veeragurunathan *et al*. (2014), various organisms such as green seaweed, brown seaweed, red seaweed, blue green algae, decapod, gastropod, bivalvia, polychaete, ophiuroidea, isopod, ectoprocta, maxillopod, ascidiacea and anthozoa were found together with *G. dura*. Thirteen species of green seaweeds, 8 species of brown seaweeds, 14 species of red seaweeds and single species of blue-green alga were found grow together with *G. edulis* cultured in Gulf of Mannar and Palk Bay, India (Ganesan *et al.* 2011; Kaliaperumal *et al.* 1993). This suggests that *Gracilaria* assemblages as one of the important habitat that supports wide range of living organisms including flora and fauna where it provides protection against tides, predators, waves and also as food sources.

## CONCLUSION

Based on morphological characteristics, three species namely *G. changii*, *G. coronopifolia* and *G. blodgettii were* found attach to net of cage culture in Santubong river and root of mangrove trees in Asajaya. In this study, it is found that *Gracilaria* able to grow both on man-made structure and natural habitat. Several aquatic organisms were found associated with *Gracilaria* assemblages such as *Acanthophora*, small crab, polychaete and bivalve. The actual percentage cover of seaweeds in Santubong and Asajaya could not be determine due to frequent collection of *Gracilaria* by the local people. Overall, the water quality in Santubong river are suitable for fisheries and mariculture while Asajaya is within normal range for mangrove in Malaysia.

## Figures and Tables

**Table 1:** Coordinate of each sampling sites

Sampling Sites	Sampling times	Brief descriptions
Santubong	November 2013, March 2014, October 2014	Located in Santubong River, near to the Santubong bridge. The cage culture is run by three local residents (En. Poli B. Pa’ee, En. Joni B. Pa’ee, En. Sudiman B. Bujang) and received guidance from Department of Fisheries Sarawak. There are 30 cages with length of 2.5 metres (m), width of 2.0 m and approximate depth of 3 m. The mesh size of the nets used is 3.5 centimetres (cm). Examples of fish culture here are grouper, red fish, yellow fish, sea bass and mud crab.
Asajaya	April 2013, March 2014, December 2014	Located in the mangrove area of Asajaya Laut village. Examples of mangrove trees observed in the area are *Rhizophora, Sonneratia* and *Avicennia*.

**Table 2:** Voucher lists for seaweed preservation.

Division	Species	Voucher number	Habitat	Location	Collection date
Rhodophyta	Order Gracilariales				
	Family Gracilariaceae				
	G. coronopifolia	SW 001-SW005	CG	Santubong	18/10/2014
	G. blodgettii	SW 006-SW010	E, M	Asajaya	27/11/2014
	G. changii	SW 011-SW015	CG	Santubong	18/10/2014

Note: CG = cage net; E = epiphyte; M = mud

**Table 3:** Summary of morphological characteristics of all *Gracilaria* found in this study.

	G. blodgettii	G. changii	G. coronopifolia
Locality	Asajaya, Sarawak	Santubong, Sarawak	Santubong, Sarawak
Type of habitat	Mangrove	Cage culture	Cage culture
Type of substrate	Mangrove root	Cage net	Cage net
Height of thallus (mm)	200	180–220	-
Colour	Dark red	Dark red	Purplish red
Type of Holdfast	Discoid	Discoid	Discoid
Constriction at base	Present	Present	Absent
Branching pattern	Secund or irregular, frequent branching	Irregular, branching occasionally	Irregular, branching shorter than previous branches
Shape of tip	Pointed	Pointed	Pointed
Diameter (mm)	1–2	1–2	1–2
No of parenchymatous layers (medulla)	3–4	3–4	3–4
No of cortical layers (cortex)	2–3	2–3	1–2

**Table 4:** Selected water quality parameters of cage culture site measured *in-situ* in Santubong and Asajaya Sarawak.

		Temperature (°C)	pH	DO (mg/L)	Salinity (PSU)	Turbidity (NTU)	Depth (m)	Transparency (m)
		
		Mean ± SD	Mean±SD	Mean±SD	Mean±SD	Mean±SD	Mean±SD	Mean±SD
Santubong	13 Nov	29.30±0.00	6.60±0.00	5.44±0.46	28.33±0.58	17.16±1.10	N/A	N/A
	14 Mar	29.90±0.00	7.82±0.06	5.29±0.07	26.00±0.00	35.10±1.85	N/A	N/A
	14 Oct	28.80±0.10	7.18±0.45	6.69±0.26	20.33±0.58	10.17±1.05	7.80±0.00	0.73±0.05
Asajaya	May 13	29.30±0.00	7.41±0.02	5.27±0.01	18.33±1.53	253.33±14.29	N/A	N/A
	Mar 14	29.37±0.06	7.80±0.01	4.05±0.03	21.00±1.73	37.63±6.60	N/A	N/A
	Dec 14	31.37±0.42	7.41±0.07	5.68±1.01	23.44±0.77	115.22±64.00	N/A	N/A

N/A: not available

**Table 5:** Selected water quality parameters of cage culture site measured *ex-situ* in Santubong and Asajaya, Sarawak.

		TSS (mg/L)	NO_2_ (mg/L)	PO_4_^3^ (mg/L)	SiO_2_ (mg/L)	Chlorophyll *a* (mg/m^3^)
		
		Mean ± SD	Mean ± SD	Mean ± SD	Mean ± SD	Mean ± SD
Santubong	13 Mar	63.33±20.42	0.012±0.001	0.053±0.02	1.070±0.286	3.037±0.254
	14 Mar	52.00±32.74	0.034±0.009	0.050±0.01	3.509±0.275	2.556±1.805
	14 Oct	27.33±2.31	0.046±0.002	0.063±0.06	0.579±0.354	7.663±0.129
Asajaya	13 May	655.57±258.91	0.015±0.00	0.160±0.03	2.740±0.090	5.937±0.663
	14 Mar	161.10±29.30	0.005±0.004	0.057±0.01	1.560±0.056	5.089±2.402
	14 Dec	234.67±38.02	0.027±0.001	0.173±0.03	1.796±0.072	3.236±0.336

**Table 6:** Comparison of water quality results in Santubong with Malaysian Marine Water Quality Criteria and Standard (MMWQCS) values.

	DO	TSS	NO_2_	PO_4_^3^
Reference value (Class 2) (mg/L)	5.00	50	0.055	0.075
Observed value (mg/L)	5.29–6.69	27.33–63.33	0.012–0.046	0.050–0.063

**Table 7:** Comparison of water quality results in Asajaya with Malaysian Marine Water Quality Criteria and Standard (MMWQCS) values.

	DO	TSS	NO_2_	PO_4_^3^
Reference value (Class E) (mg/L)	4.00	100	0.055	0.075
Observed value (mg/L)	4.05–5.68	161.10–655.57	0.005–0.027	0.057–0.173

**Table 8:** Percentage of sand, silt and clay content in Asajaya based on sampling times.

Sampling	Sediment particle size (%)						Sand (%)	Silt and clay (%)

	>1000 μm	>500 μm	>250 μm	>125 μm	>63 μm	<63 μm		
May 13	4.5	6.0	5.8	7.6	10.3	65.8	34.2	65.8
Mar 14	5.4	4.5	4.5	5.8	21.3	58.5	41.5	58.5
Dec 14	0.7	5.2	8.5	12.5	13.4	59.8	40.3	59.8
